# Factors Associated With Swallowing Function Among Physicians in Their 50s and 60s: A Cross-Sectional Study

**DOI:** 10.7759/cureus.47921

**Published:** 2023-10-29

**Authors:** Akihiko Hagiwara, Kosaku Komiya, Yuki Yoshimatsu, Ryohei Kudoh, Kazufumi Hiramatsu, Jun-ichi Kadota

**Affiliations:** 1 Department of Respiratory Medicine and Infectious Diseases, Oita University, Oita, JPN; 2 Department of Respiratory Medicine and Infectious Diseases, Oita University Faculty of Medicine, Yufu, JPN; 3 Department of Internal Medicine, University of Greenwich, London, GBR; 4 Department of Respiratory Medicine and Infectious Diseases, Oita University, Yufu, JPN

**Keywords:** conversation, aspiration pneumonia, older people, repetitive saliva swallowing test, swallowing function

## Abstract

Background: Individuals with swallowing dysfunction in their 50s and 60s may be at risk for aspiration pneumonia as they age. The association of background and lifestyle with swallowing dysfunction for those in their 50s and 60s has not been fully studied. This study aimed to clarify the relationship between lifestyle and swallowing function in this group.

Patients and methods: We targeted physicians in their 50s and 60s as participants. The repetitive saliva swallowing test (RSST) was used to evaluate swallowing function, and self-reported questionnaires about their lifestyle were administered. The associations between RSST scores and participants' backgrounds and lifestyles were analyzed.

Results: This study included 310 participants, who were divided into two groups: 162 in the low RSST group and 148 in the high RSST group. The low RSST group had significantly shorter daily conversation times and a lower incidence of hyperlipidemia than the high RSST group. On multivariate analysis, less than three hours of daily conversation time was independently related to lower RSST scores (adjusted odds ratio: 1.863; 95% confidence interval: 1.167-2.974).

Conclusions: Shorter conversation time may serve as a predictor of impaired swallowing function, potentially increasing the risk of aspiration pneumonia in the future.

## Introduction

The world’s population is gradually aging. The proportion of the global population aged 65 years or older was approximately 10% in 2022, and it is expected to reach 16% in 2050 [1]. In Japan, the population is significantly older, with people aged 65 years or older accounting for 29% of the population in 2022. This proportion is expected to reach approximately 33% in 2037 [2].

In accordance with the Japanese cause-of-death statistics, pneumonia, including aspiration pneumonia, is the fourth leading cause of death. Most of these types of pneumonia are considered aspiration pneumonia [3], which develops in patients with impaired swallowing function, including dysphagia (due to stroke or dementia) or impaired consciousness (due to alcohol, anesthesia, or sedatives), an increased chance of gastric contents reaching the lung, and an impaired cough reflex [4,5]. Swallowing video fluorography (VF) using barium and video endoscopy (VE) are widely used as gold standards for evaluating swallowing function. However, these examinations are invasive; hence, noninvasive approaches have been studied. The repetitive saliva swallowing test (RSST) is one of the noninvasive screening tests for swallowing function and was developed by Oguchi et al. and widely used in Japan [6,7]. In this test, the person is asked to swallow their saliva as many times as possible in 30 seconds, and the total number of swallows is documented as the RSST score. Based on results from a validation study, an RSST score of <3 was found to be significantly related to findings of aspiration on VF, with a sensitivity of 0.98 and a specificity of 0.66 [7].

Most cases of aspiration pneumonia occur among older people. A decline in swallowing function is unlikely to suddenly appear in this group except for an acute stroke, and it is assumed to have begun at a younger age. It has not been fully elucidated how an individual’s background or lifestyle is associated with swallowing function among people in their 50s and 60s, a time when they are younger than those susceptible to pneumonia. For example, along with direct risk factors for diseases associated with swallowing dysfunction, such as hypertension and stroke, indirect factors such as exercise, conversation, or sleep habits may be related to swallowing dysfunction [4,8,9]. By identifying these associations, we could potentially predict the future risk of aspiration pneumonia and consider interventions to reduce this risk. This study aimed to determine the factors associated with swallowing function based on not only underlying diseases but also lifestyle habits among people in their 50s and 60s.

## Materials and methods

Participants

This was a cross-sectional study using an online network platform (PLAMED Inc., Tokyo, Japan), which conducts various surveys on medical care for its members who work as physicians. We targeted 310 general physicians in their 50s and 60s, who are registered in the online network platform, as participants because the RSST could be accurately performed by these medical experts. The sample size was determined based on previous similar studies [10,11] because this was an exploratory survey and there were no previous data for sample size calculation. This study was approved by the Institutional Ethics Committee of Oita University Faculty of Medicine (approved no.: 2541; April 27, 2023). All participants gave their consent on the web screen before answering the questionnaires.

Assessment of swallowing function and data collection

The swallowing function was measured by RSST. The participants were asked to swallow their saliva as many times as possible in 30 seconds and count the number of swallows. Based on the prior studies, we set 20 times as the upper limit of the RSST score in this questionnaire. Participants were divided into two groups based on the median RSST score, and we analyzed the differences in participants’ backgrounds and lifestyles between the high and low RSST score groups. The data on age, sex, body mass index (BMI), lifestyles, comorbidities, medications, and subjective symptoms were obtained from a self-reported questionnaire created by the authors (Table 1).

**Table 1:** Survey questions

QUESTIONNAIRE
1. Please swallow your own saliva as many times as possible in 30 seconds and count the number of swallows. Answer with a number between 0 and 20.
( ) times
2. Please choose the option or answer with a number.
Age (a number between 50 and 69): ( ) years old
Sex: Male, Female, or prefer not to answer
Body mass index (kg/m^2^); <20, 20–25, 25–30, 30–35, or >35
Smoking; Current, Past, or Never
Drinking: ≧3 days/week, 1–2 days/week, or <1 day/week
Exercise (defined as >30 minutes/day); ≧3 days/week, 1–2 days/week, <1 day/week. Frequency of tooth brushing: twice or more/day, once/day, or less than once/day
Daily sleeping time: >6 hours/day, 3–6 hours/day, or <3 hours/day
Daily conversation time: >3 hours/day, 1–3 hours/day, or >1 hour/day
3. Do you have any of the following comorbidities?
Cerebrovascular disease, chronic obstructive pulmonary disease, asthma, rhinitis, gastroesophageal reflux disease, diabetes mellitus, hyperlipidemia, hypertension, head or neck tumor, neuromuscular disease, and sleep apnea syndrome
4. Do you take any of the following medications?
Antiallergen, diuretic, sedative, antidiabetic drug, antipsychotic drug, and anticholinergic drug
5. Do you have any of the following symptoms?
Nasal congestion, mouth breathing, dry mouth, snoring, phlegm, shortness of breath, near choking episodes, abnormal throat sensation, speed eating habit, difficulty in swallowing hard foods compared to 6 months ago (If you answer “Yes”, what is the reason? Swallowing, chewing, other), frequency of tooth brushing: twice or more/day, once/day, less than once/day

Statistical analysis

Statistical analyses were performed using Statistical Package for the Social Sciences (SPSS) version 22 (IBM Corp., Armonk, NY). We performed comparisons between the two groups using the Mann-Whitney U test for nonparametric continuous variables and the chi-square test or Fisher’s exact test for categorical variables. Variables presenting a p-value of <0.1 in the univariate analysis were included in the multivariate model using logistic regression analysis. A p-value of <0.05 was considered statistically significant.

## Results

Participants’ backgrounds and RSST scores

The median age of all 310 participants was 59 (interquartile range: 54-64) years, and 19 (6.1%) were female. A total of 178 (57.4%) reported a BMI of 20-25 kg/m2. RSST scores were 1-20 with a median of 12. Participants with RSST scores of 1-12 were classified as the low RSST group (n = 162, 52.3%), and those with scores of 13-20 constituted the high RSST group (n = 148, 47.7%).

Univariate analysis

The low RSST group had significantly shorter daily conversation times. In this survey, participants were asked to choose one of three answers regarding daily conversation time. The conversation time was not strictly defined whether listening time was included. Answer options were set to > three hours/day, one to three hours/day, or < one hour based on a previous study [12]. While there was no difference in “< one hour” or “≥ one hour” between the high and low RSST groups (30 (18.5%) in the low RSST group vs. 21 (14.2%) in the high RSST group; p = 0.304), a significant difference in “< three hours” or “≥ three hours” between the groups was observed (107 (66.0%) in the low RSST group vs. 75 (50.6%) in the high RSST group; p = 0.006). The high RSST group had a significantly greater incidence of hyperlipidemia (45 (30.4%) in the high RSST group vs. 32 (19.8%) in the low RSST group) (Table 2). Sleep apnea syndrome (SAS) was more commonly seen in the high RSST group, and habitual mouth breathing and near-choking episodes were more frequently reported in the low RSST group, although this was not statistically significant. BMI and the prevalence of dry mouth and underlying diseases such as cerebrovascular disease, chronic obstructive pulmonary disease (COPD), and gastroesophageal reflux disease did not differ between the two groups.

**Table 2:** Demographics Values are presented as median (interquartile range) or n (%). RSST: repetitive saliva swallowing test.

Variable	Low RSST group (n = 162)	High RSST group (n = 148)	p-value
Age (years)	59 (54–63)	60 (55–64)	0.438
Sex (female)	13 (8.0)	6 (4.1)	0.120
Body mass index (kg/m^2^)			0.106
<20	14 (8.6)	14 (9.5)	
20–25	99 (61.1)	79 (53.4)	
25–30	42 (25.9)	48 (32.4)	
30–35	3 (1.9)	7 (4.7)	
>35	4 (2.5)	0 (0)	
Drinking habits (n, %)			0.389
≥3 day/week	61 (37.7)	57 (38.5)	
1–2 day/week	31 (19.1)	20 (13.5)	
<1 day/week	70 (43.2)	71 (48.0)	
Exercise habits (n, %)			0.253
≥3 day/week	43 (26.6)	49 (33.1)	
1–2 day/week	42 (25.9)	42 (28.4)	
<1 day/week	77 (47.5)	57 (38.5)	
Smoking habits (n, %)			0.416
Current	5 (3.1)	9 (6.1)	
Past	53 (32.7)	44 (29.7)	
Never	104 (64.2)	95 (64.2)	
Comorbidities (n, %)			
Cerebrovascular disease	2 (1.2)	4 (2.7)	0.430
Chronic obstructive pulmonary disease	1 (0.6)	0 (0)	1.000
Asthma	3 (1.9)	5 (3.4)	0.486
Rhinitis	22 (13.6)	16 (10.8)	0.458
Gastroesophageal reflux disease	13 (8.0)	13 (8.8)	0.810
Diabetes mellitus	12 (7.4)	8 (5.4)	0.474
Hyperlipidemia	32 (19.8)	45 (30.4)	0.030
Hypertension	45 (27.8)	39 (26.4)	0.778
Head and neck tumor	1 (0.6)	0 (0)	1.000
Neuromuscular disease	1 (0.6)	0 (0)	1.000
Sleep apnea syndrome	3 (1.9)	9 (6.1)	0.054
Medication (n, %)			
Antiallergen	21 (13.0)	14 (9.5)	0.330
Diuretic	2 (1.2)	1 (0.7)	1.000
Sedative	13 (8.0)	14 (9.5)	0.655
Antidiabetic drug	9 (5.6)	7 (4.7)	0.743
Antipsychotic drug	0 (0)	0 (0)	
Anticholinergic drug	0 (0)	0 (0)	
Subjective symptoms (n, %)			
Nasal congestion	31 (19.1)	25 (16.9)	0.608
Mouth breathing	33 (20.4)	19 (12.8)	0.076
Dry mouth	34 (21.0)	27 (18.2)	0.544
Snoring	63 (38.9)	60 (40.5)	0.767
Phlegm	30 (18.5)	19 (12.8)	0.171
Shortness of breath	19 (11.7)	11 (7.4)	0.201
Near choking episodes	26 (16.1)	14 (9.5)	0.084
Abnormal throat sensation	7 (4.3)	2 (1.4)	0.177
Speed eating habit	79 (48.8)	70 (47.3)	0.796
Difficulty swallowing hard foods compared to 6 months ago	17 (10.5)	14 (9.5)	0.762
Frequency of tooth brushing (n, %)			0.879
≥ Twice/day	129 (79.6)	119 (80.4)	
Once/day	31 (19.1)	28 (18.9)	
< Once/day	2 (1.2)	1 (0.7)	
Sleep time (n, %)			0.266
>6 hours/day	94 (58.0)	95 (64.2)	
3–6 hours/day	68 (42.0)	53 (35.8)	
<3 hours/day	0 (0)	0 (0)	
Conversation time (n, %)			0.006
≥3 hours/day	55 (34.0)	73 (49.4)	
<3 hour/day	107 (66.0)	75 (50.6)	

Multiple logistic regression analysis

In multiple logistic regression analysis, daily conversation time of < three hours per day (adjusted odds ratio: 1.863; 95% confidence interval: 1.167-2.974) was independently related to lower RSST scores after adjustment for hyperlipidemia, SAS, mouth breathing, and near choking episodes (Table 3). This model was compatible with the Hosmer-Lemeshow test (p = 0.78).

**Table 3:** Multivariate logistic regression models for lower RSST scores RSST: repetitive saliva swallowing test.

Variable	Odds ratio	95% confidence interval	p-value
Hyperlipidemia	0.612	0.355–1.057	0.078
Sleep apnea syndrome	0.354	0.091–1.388	0.136
Mouth breathing	1.444	0.760–2.745	0.259
Near-choking episodes	1.745	0.848–3.589	0.130
Conversation time <3 hours/day	1.863	1.167–2.974	0.009

## Discussion

The current study found an association between shorter conversation time and decreased RSST scores among people in their 50s and 60s, a younger age than those who are susceptible to aspiration pneumonia. Sarcopenia has already been identified as an independent risk factor for dysphagia in community-dwelling older people [13]. The tongue plays a major role in swallowing function, and decreased tongue pressure is closely associated with dysphagia [14-16]. Reduced tongue pressure is significantly related to dysphagia on VE examination and increases the risk of pneumonia-related death among the elderly [17]. It has also been reported that those who have had singing experience for at least one year have higher RSST scores than those who do not [18]. Moreover, those who have fewer opportunities for conversation are more likely to develop dementia [19], which is another risk factor for dysphagia. Given these relationships, it is reasonable to conclude that conversation time may affect future swallowing function through the strength of oral muscles and cognitive function.

Hyperlipidemia is generally considered a risk factor for cerebrovascular and cardiovascular diseases, and it could increase the risk of dysphagia. However, the current study demonstrated that hyperlipidemia was more commonly observed in the high RSST group. This counterintuitive result can be explained by the possibility that people with better swallowing functions eat more than those without. Furthermore, cerebrovascular diseases were not associated with decreased swallowing function, although it can be considered as one of the significant risk factors of dysphagia. It is noted that the number of patients with cerebrovascular disease was small in the participants (two of 162 in the low RSST group vs. four of 148 in the high RSST group), which may have interfered with the statistics of the association between cerebrovascular diseases and swallowing function. The high RSST group had a higher incidence of SAS in the current study: nine (6.1%) in the high RSST group vs. three (1.9%) in the low RSST group. In a systematic review focusing on characteristics of obstructive SAS, the incidence of dysphagia was found to be rather high, with a range of 16%-78% [8]. Only a small percentage of participants in the current study had SAS, which could have caused random errors in the analysis. Habitual mouth breathing was more commonly observed in the low RSST group. Mouth breathing itself can lead to dry mouth, and some studies have shown that swallowing function can be influenced by oral dryness [10,11]. Furthermore, patients with COPD are more likely to have dysphagia due to the discoordination of breathing and swallowing [20,21]. Habitual mouth breathing can contribute to decreased swallowing function not only due to dry mouth but also due to the abnormal coordination of breathing.

The strength of the current study is that, to the best of our knowledge, it is the first investigation of the association between RSST scores and background or lifestyle among people in their 50s and 60s, a younger age than that of individuals susceptible to pneumonia. This study found an association between shorter conversation time and lower RSST scores, suggesting that the amount of conversation time could modify their swallowing function. Recommendations to increase conversation time might prevent the development of aspiration pneumonia in the future.

However, this study has several limitations. First, the reliability of the answers to the survey could not be measured. In particular, RSST scores were counted by the participants themselves, which made it impossible to objectively confirm whether they accurately performed the RSST. It has been reported that the mean values for RSST were 7.4 ± 1.7 (± standard deviation) with a range of 4-10 for healthy young people and 5.9 ± 2.3 with a range of 2-10 for healthy older people [6]. In a study examining RSST scores in young adults, the score was 9.6 ± 3.2 with a range of 4-18 [22]. Another study found that the RSST score in healthy people aged 41-60 years was 7.7 ± 2.5, with a range of 4-15 [10]. The RSST scores reported in the current study (median: 12) seem to be higher than those reported in these previous studies. If the results of the current study are accurate, physicians are likely to have better swallowing function than the general population. The reason for this gap could be the higher health consciousness or expert knowledge of RSST. To accurately evaluate the relationship between RSST and lifestyle, it would be better to conduct a face-to-face survey rather than an internet-based survey. Second, this study included only physicians as participants because of the presumed reliability of an accurate RSST evaluation by professionals. There might be differences in lifestyles between physicians and the general population, as noted earlier. Hence, caution is required for generalizing the results of the current study. Finally, whether the number of participants in this study is adequate for analysis remains uncertain, despite referring to similar previous studies [10,11].

## Conclusions

This cross-sectional study found that shorter conversation time was independently related to worse swallowing function, as measured by RSST, among people in their 50s and 60s. The length of conversation time may be a predictive indicator of impaired swallowing function, and it might identify a potential future risk of aspiration pneumonia. A large-scale study in the general population would be required to validate these results, and an interventional study to increase conversation time would be preferable.
